# Obstetrics Amongst the Eskimo

**Published:** 1887-02

**Authors:** H. W. Yemans

**Affiliations:** Past-Assistant Surgeon, U. S. M. H. Service, Galveston, Texas


DANIEL'S
### Texas Medical Journal.

Published Monthly.—Subscription $2.00 a Year.

Vol. 2. AUSTIN, FEBRUARY, 1887. No. 8.

### Original Articles.

☞CONTRIBUTED EXCLUSIVELY TO THIS JOURNAL.☜

*The Articles in this Department are accepted and published with the understanding that we are not responsible for, nor do we indorse the views and opinions of the writers by so doing.*

### OBSTETRICS AMONGST THE ESKIMO.

READ BEFORE THE GALVESTON MEDICAL CLUB, AND REFERRED TO DANIEL'S TEXAS MEDICAL JOURNAL FOR PUBLICATION.

*By H. W. Yemans, M. D., Past Assistant Surgeon, U. S. M. H. Service, Galveston.*

WHILE serving as Medical Officer of the U. S. Rev. Steamer, Corwin, during the annual cruise in arctic waters, in 1885, it was my good fortune to be called to assist in a case of obstetrics, which I consider well worth reporting, on account of the novel circumstances surrounding it.

Early in the morning of August 8, 1885, we came to anchor off Cape Sabine, Alaska Territory, where is a trading station of the Pacific Steam Whaling Company, in charge of the company's agent, Mr. Henry D. Woolff, through whose kindness, and by whose influence I was called in to see a native woman just on the verge of confinement, and was thus enabled to do what, as I believe, no white person has heretofore been enabled to do, viz: assist in and witness a confinement amongst the Eskimo. The day was cold and dreary, a drizling rain falling and the temperature of the atmosphere 46° F. I found the patient to be a young woman of some 20 years of age, walking about, but dressed in nothing but the lightest cotton clothing. Though I found it disagreeably chilly, she stated that she was perfectly comfortable. This was her third confinement, and the pains had begun only about one hour previous to my seeing her, which was at 9 a. m. I immediately made a digital examination and found the os about the size of a silver dime piece, with pains which gradually became more severe, occurring at intervals of about ten minutes. There was also a considerable discharge of mucus, streaked with blood—the "show" of those less distant. Dilatation steadily progressed until at 10:30 the os was about the size of a silver dollar. At this time she was removed to a smaller tent, erected in the meantime, where she and I were left alone. On one side of this tent was spread a deer-skin and on the opposite side was dug a hole about 18 inches square and 4 or 5 inches deep. Just at the edge of this hole was placed a log of wood about 6 inches in diameter, held in place by two pegs driven into the ground on either side of the ends. Immediately upon entering I was made to kneel down upon the deer-skin, and given to understand that under no circumstance must I rise to my feet. This was afterwards explained to me as absolutely essential to the future welfare of all concerned. My companion maintained the kneeling or sitting position during the entire time of my stay there, neither standing up nor lying down once. I had but time to make another digital examination, when I found the os still more dilated and the membranes bulging considerably, when the "waters" broke, which was announced by those waiting on the outside by a loud yell, immediately followed by the advent of the girl's mother, with a bag of plucked rein-deer hair in her hand, with which she at once proceeded to line the hole previously mentioned—maintaining meanwhile the kneeling position. As soon as this was done the old woman left, and a "showman" or Eskimo "medicine-man" soon after took up a kneeling position at the entrance to the tent, gathered the flops about his face and sang, in a low bass voice, a really pretty song, of some two or three minutes length. This done, the young woman removed her drawers and took up her position over the hole mentioned, her toes resting on the ground on one side and her knees widely separated on the log on the opposite side. She supported herself by placing her hands on her knees and immediately began to "bear down" with "all her might and main," her groans being answered by sharp cries to "bear down hard" from the old women and girls surrounding the tent. Desiring to obtain all the information possible, I allowed her to pursue her own course of procedure. I remaining a silent, but decidedly interested and observant spectator. Pains now occurred in rapid succession, she bearing down all the time, and occasionally making pressure over the abdomen, stimulated by the cries and commands of those without. It was now 11:15, and in another 30 minutes the child was born. For the ten minutes only preceding its escape did the head press against the perineum; the body following the head almost immediately. At once having prepared myself beforehand, I jumped to my feet, and tied and cut the cord almost before she had time to communicate the fact to those on the outside, which brought forth a chorus of yells and screams of remonstrance. Very little hemorrhage occurred, and the mother turning for a moment to ask me whether it was an Ag-a-nar-ok or Ex-lily-gok, (girl or boy) began again to bear down and press with her folded arms the abdomen to expel the secundines. Her efforts were futile, however, which fact she told those in waiting, who told her to insert her finger into her throat and "gag" herself. This she did repeatedly for several minutes, the little fellow—it was a boy—meanwhile crying lustily from his bed of hair. Sympathizing with her in her vain efforts I asked, after waiting some time, if I should assist her, to which she readily assented and I, without difficulty, extracted the placenta and membranes. This accomplished, she spoke to those on the outside, who brought a tub of warm water which they pushed under the tent, beside which she seated herself and requested me to hand her her child, which I did. She then bathed, using soap, in quite a civilized fashion. This last procedure resulted from her intercourse with the whites, it being usually performed with urine, to which all contribute their share beforehand, being the fluid generally used for this purpose.

I learned afterwards that they looked upon me with not a little interest, and have inquired since concerning my welfare, as to come into contact with anything connected with the child (blood, secundines, &c.) by any one but the mother, (who must bury them all) means certain death, at no distant day, to the person thus made "unclean."

Four days later I again saw the mother and child, when both were doing well, excepting caked breast and sore nipples, from which the mother suffered. One year has since elapsed, and I am told by those coming from that region that both are very well indeed.

I desire to supplement the foregoing by the recital of a few additional interesting facts not observed by me, but which I am convinced are correct.

After a child is born and the after birth comes away, and not until then, is the cord tied, and then always by the mother, she using a small piece of flint or other hard stone for the purpose, which the child wears in a bag hung about its neck, as a charm, during its entire after life.

Births and deaths are not allowed to occur under any circumstances in the houses used as dwellings, but should such an event by any accident occur, the place is immediately deserted and never entered again. Temporary structures, even in mid-winter, are erected for those about to be confined, or who are considered as apt to die, to which they are removed, and in most cases left entirely alone.

In cases of child-birth the mother remains in this temporary structure four days, if a boy, and five days if a girl, the woman's mother or other female relative, conveying the necessary food to the mother. The child is immediately put to the breast after delivery, washing, tying the cord, &c.

Deaths of either mother or child are rare, so I am informed, and confinements are very seldom attended with any considerable difficulty.

First menstruations occur at from twelve to fourteen years of age, and for one year afterwards the girl is not allowed to play with other children, or to go upon either water or ice; like prohibition occurring during the menstruation of any woman.

It will thus be seen that pubesence occurs amongst these people at a much earlier age than is given by most authorities, and according to those who *ought* to know what naturally follows, is not of the cold, passionless description as we are usually told. Those who have wintered there tell me that entire days and nights are frequently spent in the wildest of sensual debauches.

These people know nothing of medication, except in cases of constipation, when copious draughts of seal oil are taken, they depending entirely upon "showmanism," which is nothing more nor less than witchcraft. In cases of obstinate disease, the person afflicted is made to fast for four or five days, and is then made to eat a piece of human flesh, coming from some person accidently or otherwise killed.

If three or four children die before reaching the age of five years, those born afterwards are thrown away and allowed to perish immediately after birth; the same being done if the first three or four children happen to be boys.

